# Impact of Anthelminthic Treatment in Pregnancy and Childhood on Immunisations, Infections and Eczema in Childhood: A Randomised Controlled Trial

**DOI:** 10.1371/journal.pone.0050325

**Published:** 2012-12-07

**Authors:** Juliet Ndibazza, Harriet Mpairwe, Emily L. Webb, Patrice A. Mawa, Margaret Nampijja, Lawrence Muhangi, Macklyn Kihembo, Swaib A. Lule, Diana Rutebarika, Barbara Apule, Florence Akello, Hellen Akurut, Gloria Oduru, Peter Naniima, Dennison Kizito, Moses Kizza, Robert Kizindo, Robert Tweyongere, Katherine J. Alcock, Moses Muwanga, Alison M. Elliott

**Affiliations:** 1 Medical Research Council/Uganda Virus Research Institute Uganda Research Unit on AIDS, Entebbe, Uganda; 2 Entebbe Hospital, Entebbe, Uganda; 3 Department of Psychology, Lancaster University, Lancaster, United Kingdom; 4 Uganda Virus Research Institute, Entebbe, Uganda; 5 School of Veterinary Medicine, Makerere University, Kampala, Uganda; 6 London School of Hygiene and Tropical Medicine, London, United Kingdom; Liverpool School of Tropical Medicine, United Kingdom

## Abstract

**Background:**

Helminth infections may modulate immune responses to unrelated pathogens and allergens; these effects may commence prenatally. We addressed the hypothesis that anthelminthic treatment in pregnancy and early childhood would improve responses to immunisation and modulate disease incidence in early childhood with both beneficial and detrimental effects.

**Methods and Findings:**

A randomised, double-blind, placebo-controlled trial was conducted in Entebbe, Uganda [ISRCTN32849447]. In three independent randomisations, 2507 pregnant women were allocated to receive single-dose albendazole or placebo, and praziquantel or placebo; 2016 of their offspring were randomised to receive quarterly single-dose albendazole or placebo from age 15 months to 5 years. Primary outcomes were post-immunisation recall responses to BCG and tetanus antigens, and incidence of malaria, diarrhoea, and pneumonia; incidence of eczema was an important secondary outcome. Analysis was by intention-to-treat. Of 2345 live births, 1622 (69%) children remained in follow-up at age 5 years. 68% of mothers at enrolment, and 11% of five-year-olds, had helminth infections. Maternal hookworm and *Schistosoma mansoni* were effectively treated by albendazole and praziquantel, respectively; and childhood hookworm and *Ascaris* by quarterly albendazole. Incidence rates of malaria, diarrhoea, pneumonia, and eczema were 34, 65, 10 and 5 per 100 py, respectively. Albendazole during pregnancy caused an increased rate of eczema in the children (HR 1.58 (95% CI 1.15–2.17), p = 0.005). Quarterly albendazole during childhood was associated with reduced incidence of clinical malaria (HR 0.85 (95% CI 0.73–0.98), p = 0.03). There were no consistent effects of the interventions on any other outcome.

**Conclusions:**

Routine use of albendazole in pregnancy may not always be beneficial, even in tropical developing countries. By contrast, regular albendazole treatment in preschool children may have an additional benefit for malaria control where helminths and malaria are co-endemic. Given the low helminth prevalence in our children, the effect of albendazole on malaria is likely to be direct.

**Trial registration:**

Current Controlled Trials ISRCTN32849447

## Introduction

It has been estimated that 2 billion people are infected with schistosomes and soil-transmitted helminths (STH) [1] with up to one third of the population of Sub-Saharan Africa affected by STH infections. [2] Globally, malaria, diarrhoea and pneumonia are the commonest causes of morbidity and mortality in childhood, and together they account for nearly 50% of all under-five deaths in Africa. [3] Tuberculosis incidence is estimated at over 9 million new cases per year, with 1.8 million deaths, [4] and the efficacy of Bacille Calmette Guérin (BCG) immunisation against tuberculosis is poor in tropical latitudes. [5] The geographic overlap between helminths and such important infectious agents has led to the immunologically plausible hypothesis that helminth infections influence the epidemiological patterns of other diseases. [6]

Helminths induce potent type 2 and regulatory immune responses, both of which may oppose the type 1 responses required for protection against other pathogens, or by vaccines, [7] thus increasing susceptibility to infectious diseases either directly, or through diminution of vaccine effectiveness. Conversely, down-regulation of unwanted inflammatory responses during chronic helminth infection may have benefits. Contrasting effects of helminths on susceptibility to malaria infection, and on the damaging inflammatory sequelae, may explain in part the conflicting results of previous studies on helminth-malaria interactions, some of which suggest detrimental, and some beneficial, effects. [8] Benefits may also include the prevention of allergy and autoimmunity, [9] which are rare in tropical, developing countries. [10] Prenatal exposure to helminths may be particularly important: *in utero* exposure to maternal helminths has long term implications for the child's response to related worm infections [11] but whether such immunological effects have a measurable impact on efficacy of unrelated vaccines [12] or on incidence of unrelated infectious diseases, and whether these effects may be reversed by the administration of anthelminthics during pregnancy and early childhood, is less clear. Helminth infections in early childhood may also have important effects on the responses generated as children undergo their initial exposures to infections and allergens.

We established the Entebbe Mother and Baby Study to investigate effects of helminths and their treatment during pregnancy and early childhood on immune responses to vaccines and on susceptibility to infectious and allergy-related diseases in early childhood. [13] Findings in infancy showed little effect of maternal helminths, or of their treatment during pregnancy, on vaccine and infectious outcomes, [14], [15] but significant adverse effects of maternal anthelminthic treatment on infantile eczema. [16] We have now investigated the effects of quarterly albendazole versus placebo in preschool children, as well as the longer-term effects to age 5 years of the intervention in pregnancy, on the recall response to BCG and tetanus immunisation given in infancy, on incidence of infectious diseases (malaria, diarrhoea and pneumonia) and eczema, and on other outcomes for which anthelminthic therapy has been proposed to be beneficial: anaemia, growth and cognitive development. [17]

## Materials and Methods

### Study design and participants

As previously described, the study, based at Entebbe General Hospital, Uganda, was a trial with three randomised, double-blind, placebo-controlled interventions at two times, in a 2×2(×2) factorial design: women were randomised to albendazole versus placebo and praziquantel versus placebo during pregnancy; their children were randomised to quarterly albendazole versus placebo from age 15 months to 5 years [ISRCTN32849447]. [13]

Written informed consent was obtained twice: from the mother during pregnancy, and from the mother or caregiver when the child reached age one year, or the first subsequent visit, for the trial of treatment during childhood. The study was approved by the Science and Ethics Committee of the Uganda Virus Research Institute, the Uganda National Council for Science and Technology and the ethics committee of the London School of Hygiene and Tropical Medicine. The protocol for this trial and supporting CONSORT checklist are available as supporting information; see Checklist S1 and Protocol S1.

### Randomisation and masking

Randomisation codes were generated by the trial statistician using Stata version 7 (College Station, Texas, USA), with numbers allocated in blocks of 100 and 80 to the mother and child treatment groups, respectively; all three randomisations were independent of each other.

Inclusion and exclusion criteria for the women, and intervention and randomisation procedures during pregnancy have been described in detail. [15] Briefly, healthy pregnant women from the study area, and planning to deliver in Entebbe Hospital, pregnant women were assigned in a 1∶1∶1∶1 ratio to receive simultaneously single-dose albendazole (400 mg) and praziquantel (40 mg/kg), or albendazole and a praziquantel-matching placebo, or an albendazole-matching placebo and praziquantel, or an albendazole-matching placebo and a praziquantel-matching placebo (albendazole and matching placebo: GlaxoSmithKline, Brentford, UK; praziquantel tablets, (Medochemie Ltd, Limassol, Cyprus) were used to prepare identical praziquantel 300 mg and placebo capsules (Almedica Europe Ltd, Deeside, UK)). The intervention was given under direct observation, during the second or third trimester of pregnancy.

When children made their first quarterly visits from age 15 months onwards, they were randomised in a 1∶1 ratio to receive quarterly albendazole or placebo. All children of participating mothers were eligible for inclusion. From age 15 to 21 months, children received syrups (5 ml) equivalent to 200 mg albendazole or matching placebo, labelled according to the randomisation code by the manufacturer. From age 2 to 5 years, children received chewable tablets of albendazole (400 mg) or matching placebo, packaged and sealed by an independent committee of Medical Research Council staff in Entebbe, not otherwise involved in the study, into consecutively numbered envelopes according to the randomisation code. Trained study nurses allocated the numbers sequentially and gave the quarterly intervention, observing that it was taken correctly. All participants and study staff were blinded to the drug allocation throughout the trial.

### Procedures

Women provided a single stool sample prior to randomisation and following delivery; all received anthelminthic treatment six weeks after delivery. Their children were followed up for routine immunisations, and then quarterly, to age 5 years. Children received BCG and oral polio immunisations at birth, polio, diphtheria, pertussis, tetanus, hepatitis B and *Haemophilus influenzae* type B immunisations at 6, 10, and 14 weeks, and measles immunisation at 9 months. At routine annual visits, blood and stool samples were collected, and growth outcomes recorded. Irrespective of their allocated intervention group, children found to have helminth infections on examination of the annual stool sample were treated as indicated for the species identified.

Primary outcomes were immune response at age 5 years to BCG and tetanus immunisation, and incidence of malaria, diarrhoea, pneumonia, measles, and tuberculosis during childhood. Following evidence from our preliminary study of a possible effect of maternal helminths and of albendazole treatment during pregnancy on infantile eczema, [18] allergy-related disease events were added as an important secondary outcome for the main trial. [13] Other secondary outcomes were growth and anaemia assessed at routine annual visits, and cognitive development. We also considered asymptomatic *Plasmodium falciparum* parasitaemia recorded at routine annual visits, as an unplanned exploratory outcome.

Immunological assays were simplified compared to the original protocol, for logistic and cost reasons. The recall response to vaccines given during infancy was assessed at 5 years using cytokine responses to crude culture filtrate proteins (cCFP) of *Mycobacterium tuberculosis*, and antigen 85 (a major secreted protein complex that exhibits cross-reactivity between many mycobacterial species), and to tetanus toxoid, as indicators of response to BCG and tetanus immunisation. We examined stimulated interferon-γ (type 1), interleukin-5 and interleukin-13 (type 2), and interleukin-10 (regulatory) responses in a whole-blood assay, as previously described [19]. Total serum anti-tetanus IgG was measured by ELISA. [15]

Illness outcomes were detected prospectively when sick children were brought to the clinic for treatment. Malaria was diagnosed as fever (≥37.5°C) with any parasitaemia (whether asexual forms or gametocytes); diarrhoea was diagnosed based on the mother's definition; [20] pneumonia was defined as cough with difficulty in breathing, and fast breathing (defined by age), with or without abnormal breath sounds; [21] measles was defined by standard clinical criteria confirmed by measurement of specific antibody; [22] children with suspected tuberculosis were investigated as clinically indicated; [23] eczema was defined as a recurrent itchy rash with either wet, weeping skin or dry, scaly skin, and with a typical distribution. One study doctor underwent specialised training in the diagnosis of skin conditions at the National Referral and Teaching hospital in Uganda, and trained other doctors at the study clinic, to ensure accurate and consistent definition of eczema. For development outcomes, children were assessed at age 5 years at the study clinic for cognitive ability, executive function and motor ability using 13 measures created or adapted for the study (see Text S1 and Table S1). [24]

Haemoglobin was estimated by Coulter analyzer (Beckman Coulter AC-T 5 diff CP; Beckman Coulter, Nyon, Switzerland). Leishman stained thick blood films were examined for *P. falciparum* ring forms or gametocytes. The modified Knott's method was used to examine for microfilariae. [25] Stools were examined for helminth ova using the Kato-Katz method: [26] two slides were prepared from each sample; slides were read within 30 minutes for hookworm ova, and the following day for other species. Stools were cultured for Strongyloides. [27] Urine examination for *S. haematobium* was not conducted because it is rare in this setting. [28] Quality control for haematology and malaria parasitology was provided through the United Kingdom National External Quality Assessment Schemes, and for Kato Katz analyses through the Vector Control Programme of the Ministry of Health, Uganda, with consistently good results. HIV status was determined for mothers at enrolment during pregnancy, and for children aged 18 months or above, using a rapid antibody test algorithm; for infants RNA and DNA polymerase chain reaction methods were used. [15]

Serious adverse events were defined as any clinical event considered by the clinician to be severe or life-threatening, or that resulted in death. This included events requiring unexpected hospitalisation or prolongation of hospitalisation, or resulting in persistent or significant disability or incapacity, but not hospitalisations expected in this setting for illnesses such as malaria. Pre-specified serious adverse events include miscarriage, stillbirth, neonatal death, major congenital abnormality, maternal death during the puerperium, death of the child at any time and anaphylaxis, severe acute bronchospasm or seizures within 24 hours of administration of the study drug.

### Statistical analysis

Data were analysed after all children had reached age 5 years. Data from routine annual visits were included if the child attended within 1 month before and 2 months after their birthday. Results for all twins and triplets were included. All analysis was by intention-to-treat.

The cohort size of 2500 was calculated to give 80% power to detect treatment effect sizes at p<0.05. For recall responses to immunisation, allowing for anticipated loss to follow-up, samples from 1046 children assessed at 5 years would detect differences in cytokine responses of 0.14 log_10_ between maternal or childhood intervention groups, assuming a standard deviation of 0.80 log_10_.

For illness events, a detrimental effect of anthelminthic treatment was anticipated for malaria and eczema, and a beneficial effect for other infections. For the childhood intervention, estimated effect sizes that could be detected were rate ratios of 1.07, 0.94, 0.81 and 1.30 for malaria, diarrhoea, pneumonia and eczema, assuming rates in the placebo group of 80, 100, 10 and 5 per 100 person-years, respectively. The study had power to detect slightly smaller long-term effects of the maternal interventions, since the follow-up time (from birth to 5 years) was longer. The incidence of both tuberculosis and measles was expected to be low, therefore only very large differences in rates would be detected.

To evaluate the effect of the childhood intervention on the prevalence of each helminth, we combined data from all annual visits, examining the overall effect of each treatment using generalised estimating equation (GEE) logistic regression models to calculate odds ratios (ORs) allowing for within-child correlations.

Cytokine and antibody responses showed skewed distributions, some with disproportionate numbers of zero values. Results were transformed to log_10_(concentration+1) and analysed by linear regression with bootstrapping to estimate bias-corrected accelerated confidence intervals. [29] Regression coefficients were back-transformed to give geometric mean ratios.

For long-term effects of maternal interventions on incidence of diseases in childhood, time at risk began at birth. For effects of the childhood intervention, sample size calculations assumed time at risk from age 1 to 5 years. However, recognising that the intervention actually commenced when the child took the first intervention dose, the analysis plan was modified such that time at risk began at the date of randomisation and receipt of the first dose (age 15 months, or later if the child missed the 15-month visit). For both analyses, time at risk was censored at loss to follow-up, death or age 5 years. All children were included until censoring, regardless of whether or not they had made a clinic visit for illness. For each disease, we calculated incidence rates for all events. Episodes within 14 days of an initial presentation with the disease were considered to be part of the same episode and excluded from the analysis; time at risk was adjusted accordingly. Hazard ratios (HRs) for the effect of treatment on all-events disease incidence were calculated using Cox regression with robust standard errors to allow for within-child clustering. The prevalence of asymptomatic malaria parasitaemia at each annual visit was compared between treatment groups using logistic regression.

Sex-specific z-scores for weight-for-age, height-for-age and weight-for-height at 5 years were derived from WHO growth standard reference scales, using WHO Anthro version 3 for children measured when aged less than 5 years and 1 month and WHO AnthroPlus version 3 for children who were measured more than 1 month (and less than 2 months) after their fifth birthday. We examined the effects of the interventions on the continuous z-scores and on haemoglobin at each annual visit using linear regression. We also combined data from all annual visits, examining the overall effect of each treatment using GEE linear regression models to allow for within-child correlations. Effects of the interventions on tests of motor and cognitive functioning at 5 years were examined using linear regression.

For the maternal interventions, two pre-specified subgroup analyses were performed, examining effects of albendazole treatment in children of mothers with hookworm infection, and effects of praziquantel treatment in children of mothers with schistosomiasis. Differences between subgroups were examined by fitting interaction terms in regression models. For the childhood intervention we conducted a post-hoc subgroup analysis of malaria incidence by age group, fitting an interaction term to test for effect modification. Interactions between the childhood intervention and each maternal intervention were also examined by fitting interaction terms in the regression models.

All p-values are two-sided with no adjustment made for multiple comparisons. Data were analysed using Stata version 11, except for developmental scores, for which SPSS version 16.0 was used.

## Results

**Figure 1:**
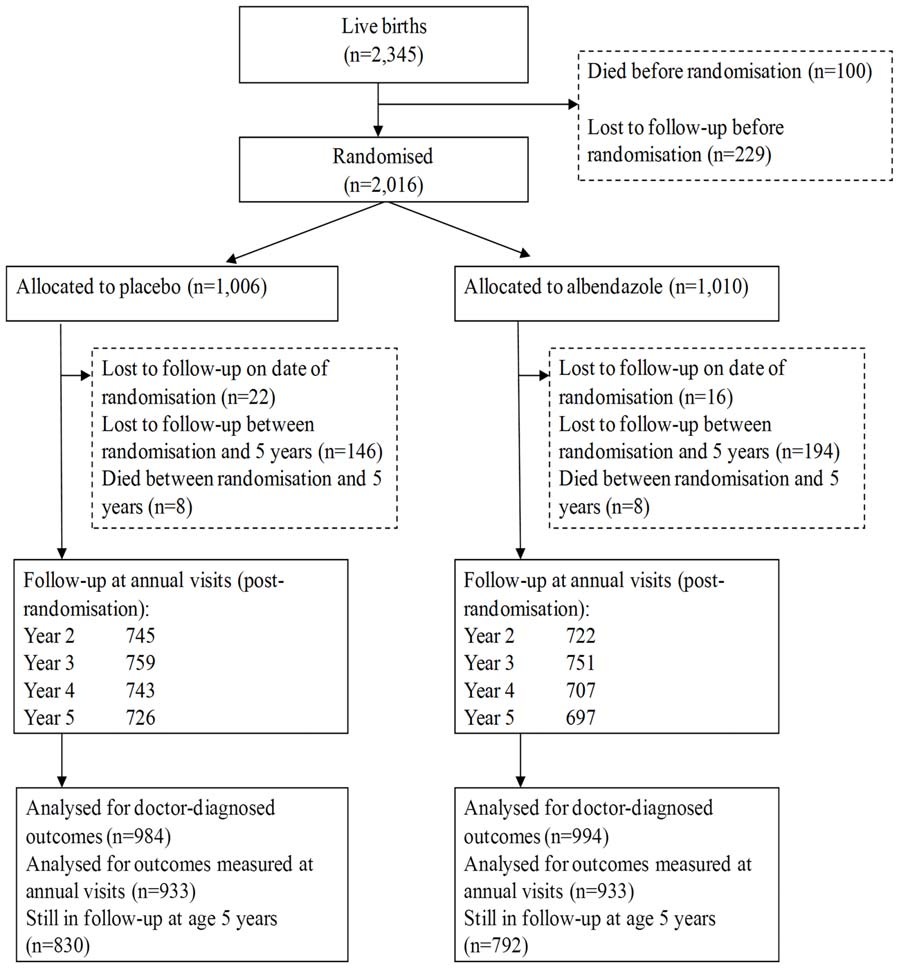
Flow of participants through the childhood trial.

2507 women were enrolled between April 2003 and November 2005. Their offspring were followed from birth to age 5 years, the planned end of the trial. There were 2345 live-born children of whom 2016 were later randomised into the childhood intervention trial, with 1622 remaining in follow-up at age 5 years (69%). The trial profile for the maternal intervention up to the end of infancy has been published previously. [15] The trial profile for the childhood intervention is shown in Figure 1; follow-up was slightly lower in the albendazole group compared to the placebo group with numbers of children still under follow-up at 5 years of 792 and 830, respectively.

Baseline characteristics were comparable between the four treatment groups in pregnancy (Table S2), [15] and between the two treatment groups in childhood, with the exceptions that maternal hookworm was slightly less prevalent, and eczema prior to the childhood intervention slightly commoner, among children allocated to quarterly albendazole than among those allocated to placebo (Tables 1 and 2). Mothers of children who participated in the childhood intervention were on average slightly older, less likely to be primigravidae, and less likely to have asymptomatic *P. falciparum* infection than mothers of children who did not participate; children who participated were on average heavier at birth, and less likely to be HIV positive or to have asymptomatic *P. falciparum* at one year of age than those who did not. There was no evidence of interaction between maternal and childhood treatments for any outcome, therefore the effects of each treatment were examined independently.

**Table 1:** Baseline characteristics of mothers whose children were children enrolled in the trial of quarterly albendazole versus placebo from age 15 months to five years.

		Albendazole Placebo	Albendazole
Number of mothers with children randomised^a^		994	1002
Age in years, mean ± SD		23.95±5.38	23.76±5.43
Education (4 mv)^b^			
	None	36 (4%)	33 (3%)
	Primary	498 (50%)	509 (51%)
	Secondary	381 (38%)	362 (36%)
	Tertiary	79 (8%)	94 (9%)
Household socioeconomic status (41 mv)^c^			
	(low) 1	48 (5%)	67 (7%)
	2	77 (8%)	88 (9%)
	3	320 (33%)	289 (30%)
	4	280 (29%)	281 (29%)
	5	190 (19%)	203 (21%)
	(high) 6	62 (6%)	50 (5%)
Gravidity			
	1	247 (25%)	265 (26%)
	2–4	582 (59%)	571 (57%)
	≥5	165 (17%)	166 (17%)
Trimester at treatment (3 mv)			
	2	512 (52%)	506 (51%)
	3	482 (48%)	493 (49%)
Maternal history of asthma (1 mv)		19 (2%)	22 (2%)
Helminth infections			
	Hookworm (7 mv)	459 (46%)	409 (41%)
	*S. mansoni* (7 mv)	189 (19%)	177 (18%)
	*M. perstans* (8 mv)	222 (22%)	201 (20%)
HIV positive		109 (11%)	94 (9%)
Malaria parasitaemia (36 mv)		104 (11%)	93 (9%)
Owns mosquito net (3 mv)		494 (50%)	519 (52%)
Intermittent preventive treatment for malaria during pregnancy (91 mv)			
	1 dose	230 (24%)	209 (22%)
	2 doses	626 (66%)	654 (68%)
	≥3 doses	87(10%)	99 (10%)
Maternal tetanus immunisation during pregnancy			
	0 doses	220 (22%)	231 (23%)
	1 dose	600 (60%)	592 (59%)
	≥2 doses	174 (18%)	179 (18%)
Maternal treatment			
	Albendazole+praziquantel	244 (24%)	256 (26%)
	Albendazole placebo+praziquantel	247 (25%)	249 (25%)
	Albendazole+praziquantel placebo	247 (25%)	257 (26%)
	Double placebo	256 (26%)	240 (24%)

a The number of mothers with children randomised is lower than the number of children randomised due to 20 sets of twins.

b mv: missing values.

c Household socioeconomic status was scored based on building materials of the home, number of rooms and items owned, “1” representing lowest and “6” representing highest status.

**Table 2:** Characteristics of children enrolled in the trial of quarterly albendazole versus placebo from age 15 months to five years, at the time of randomisation.

		Albendazole placebo	Albendazole
Number of children randomised^a^		1006	1010
Age at randomisation in years, mean ± SD		1.52±0.54	1.52±0.50
Male		531 (53%)	510 (51%)
Birthweight (362 mv^b^), mean ± SD		3.19±0.48	3.16±0.50
HIV status			
	Unexposed	894 (89%)	914 (90%)
	Exposed, uninfected	88 (9%)	79 (8%)
	Exposed, infected	18 (2%)	13 (1%)
	Exposed, unknown	6 (0.6%)	4 (0.4%)
P.falciparum at first annual visit (394 mv)		41 (5%)	51 (6%)
Any worm infection at first annual visit (536 mv)		19 (3%)	22 (3%)
Received deworming elsewhere during infancy (356 mv)		132 (16%)	161 (20%)
Number of clinic visits for illness before randomisation, mean ± SD		6.59±3.51	6.52±3.44
Any clinic visit pre-randomisation for:			
	Malaria	361 (36%)	337 (33%)
	Diarrhoea	747 (74%)	765 (76%)
	Pneumonia	193 (19%)	184 (18%)
‵	Eczema	68 (7%)	98 (10%)

a The number of mothers with children randomised is lower than the number of children randomised due to 20 sets of twins;

b mv: missing values;

Following the childhood randomisation, adherence was slightly lower in the albendazole group than in the placebo group, reflecting the slightly lower follow-up in the albendazole group: mean (SD) doses received (of a maximum of 16) was 11.8 (4.5) for placebo versus 11.4 (4.1) for albendazole, p = 0.08; 22% versus 19% of participants received all 16 doses, respectively (p = 0.05). At each annual visit, between 20% and 30% of children were reported to have received anthelminthic treatment elsewhere. Over the four years of the childhood trial, 81% of those in the placebo group and 83% of those in the albendazole group reported receiving anthelminthic treatment elsewhere at least once.

At enrolment, 68% of women were infected with at least one helminth species, [15] but prevalence was low among children at all annual visits: 3.6%. 5.6%. 8.9%. 11.0% and 10.5% at 1, 2, 3, 4 and 5 years, respectively.. At age 5 years, 5.5% of children were infected with *Trichuris trichiura*, 2.3% with *Schistosoma mansoni,* 1.1% with *Ascaris lumbricoides,* 1.0% with *Hymenolepis nana*, 0.5% with hookworm, and 0.2% with *Mansonella perstans* (Table S3). Combining data from all annual visits, the childhood intervention was associated with a reduction in prevalence of *A. lumbricoides* and hookworm (OR 0.41, 95% CI: 0.23–0.71, p = 0.001 and OR 0.47, 95% CI: 0.23–0.96, p = 0.04, respectively) but no other helminth infection was affected (Table 3).

**Table 3:** The prevalence of helminth infection at each routine annual visit, by childhood treatment group.

		Age			
Helminth	Childhood Treatment	2 years (n = 1428)^1^	3 years (n = 1429)^1^	4 years (n = 1366)^1^	5 years (n = 1319)^1^
*Trichuris trichiura*	Placebo	10 (1.4%)	32 (4.4%)	40 (5.8%)	39 (5.8%)
	Albendazole	16 (2.3%)	31 (4.4%)	35 (5.2%)	34 (5.3%)
*Ascaris lumbricoides*	Placebo	14 (1.9%)	19 (2.6%)	11 (1.6%)	11 (1.6%)
	Albendazole	7 (1.0%)	6 (0.9%)	6 (0.9%)	3 (0.5%)
*Schistosoma mansoni*	Placebo	3 (0.4%)	5 (0.7%)	9 (1.3%)	20 (3.0%)
	Albendazole	6 (0.9%)	7 (1.0%)	10 (1.5%)	10 (1.6%)
Hookworm	Placebo	5 (0.7%)	7 (1.0%)	10 (1.4%)	2 (0.3%)
	Albendazole	2 (0.3%)	1 (0.1%)	5 (0.7%)	3 (0.5%)
*Hymenolepis nana*	Placebo	3 (0.4%)	7 (1.0%)	10 (1.5%)	7 (1.0%)
	Albendazole	3 (0.4%)	7 (1.0%)	7 (1.0%)	6 (1.0%)
*Mansonella perstans*	Placebo	2 (0.3%)	2 (0.3%)	2 (0.3%)	1 (0.1%)
	Albendazole	1 (0.1%)	2 (0.3%)	3 (0.4%)	2 (0.3%)
*Trichostrongylus*	Placebo	1 (0.1%)	1 (0.1%)	1 (0.1%)	0 (0%)
	Albendazole	2 (0.3%)	0 (0%)	0 (0%)	0 (0%)

1 For each annual visit, denominators are slightly lower than in Web Table 3 due to excluding children who were first randomised at that annual visit.

At age 5 years cytokine responses to mycobacterial antigens were assessed among 1190 children who had received BCG immunisation at Entebbe Hospital. Cytokine and antibody responses to tetanus toxoid were assessed, respectively, among 1162 and 1129 children who had received all three doses of tetanus immunisation. The proportion of children for whom positive responses to cCFP, antigen 85 and tetanus toxoid were detected varied by cytokine: for cCFP, 86%, 48%, 71% and 90% of infants had positive responses to IFN-γ, IL-5, IL-13 and IL-10, respectively. Corresponding numbers for antigen 85 were 76%, 40%, 63% and 81%, respectively, and for tetanus toxoid the figures were 39%, 39%, 60% and 52%, respectively. There was no effect of anthelminthic treatment during pregnancy on these responses, either overall, or in the pre-specified sub-group analyses (data not shown). Quarterly treatment with albendazole was associated with somewhat lower IFN- γ, IL-5 and IL-13 responses to cCFP (with strongest effects for IFN- γ and IL-13), but no similar effect was seen on responses to antigen 85 or to tetanus toxoid, and there was no effect on anti-tetanus antibody levels (Table 4).

**Table 4:** The effect of quarterly albendazole during childhood on the recall response to mycobacterial and tetanus antigens, and on anti-tetanus antibody levels, at age 5 years.

		Intention-to-treat analysis		
Antigen	Cytokine/antibody	Geometric mean^a^		Geometric mean ratio (95% CI)^b^
		Albendazole Placebo	Albendazole	
		n = 616	n = 574	
**cCFP**	**Interferon-** γ **(pg/ml)**	195	141	0.73 (0.56, 0.96)
	**IL-5 (pg/ml)**	6.2	5.3	0.86 (0.69, 1.05)
	**IL-13 (pg/ml)**	30	22	0.71 (0.55, 0.94)
	**IL-10 (pg/ml)**	51	51	1.00 (0.82, 1.20)
		n = 616	n = 574	
**Antigen 85**	**Interferon-** γ **(pg/ml)**	54	50	0.94 (0.72, 1.27)
	**IL-5 (pg/ml)**	4.1	4.3	1.05 (0.84, 1.28)
	**IL-13 (pg/ml)**	16	14	0.84 (0.64, 1.09)
	**IL-10 (pg/ml)**	24	23	0.96 (0.80, 1.18)
		n = 597	n = 565	
**Tetanus toxoid**	**Interferon-** γ**(pg/ml)**	4.4	5.6	1.27 (0.97, 1.62)
	**IL-5 (pg/ml)**	4.2	4.4	1.04 (0.85, 1.35)
	**IL-13 (pg/ml)**	15	16	1.10 (0.85, 1.49)
	**IL-10 (pg/ml)**	5.0	4.8	0.97 (0.81, 1.19)
		n = 579	n = 550	
**Tetanus toxoid**	**Total IgG (mIU/ml)**	129	122	0.95 (0.76, 1.18)

cCFP: crude culture filtrate proteins of *Mycobacterium tuberculosis*.

a geometric mean of response concentration +1;

b bias-corrected accelerated confidence intervals computed by bootstrapping.

**Figure 2:**
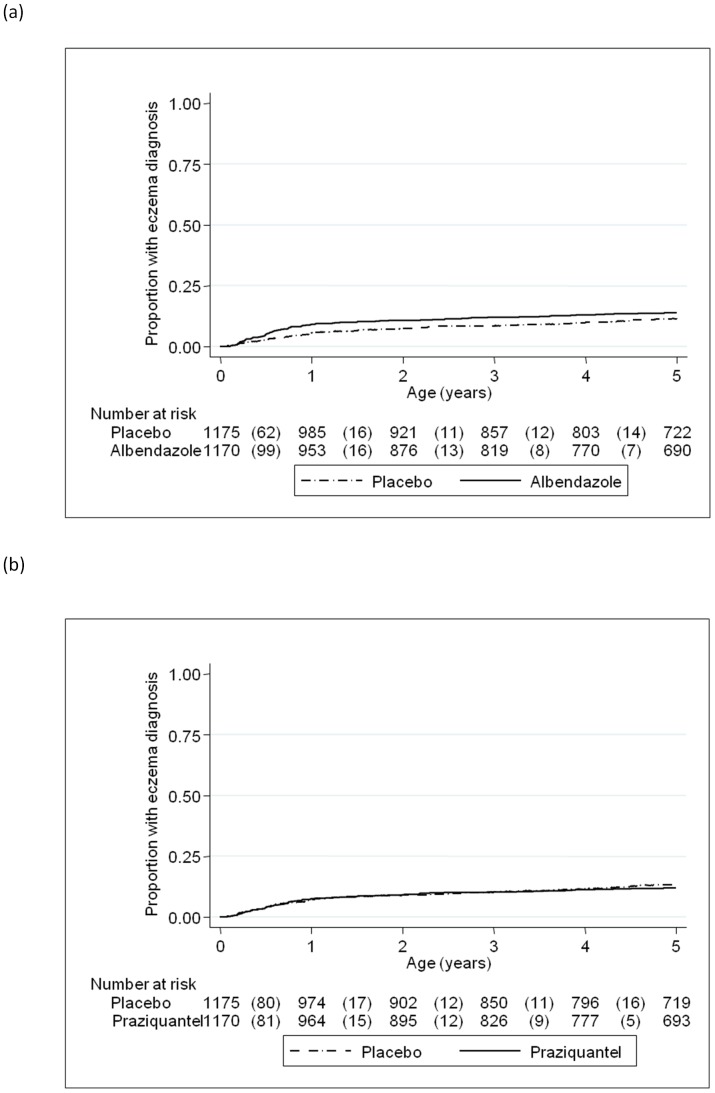
Effect of anthelminthic treatment during pregnancy on eczema incidence in the children. Kaplan-Meier survival estimates for time to first (or only episode) of eczema (a) comparing children whose mothers received albendazole during pregnancy with those whose mothers received albendazole-placebo (b) comparing children whose mothers received praziquantel during pregnancy with those whose mothers received praziquantel-placebo. Numbers shown in the tables are number of events (in brackets) and number of children at risk.

From birth to 5 years, a total of 33,178 clinic visits for illness were made; numbers of visits were similar across both maternal and childhood treatment groups (data not shown). All-event incidence rates for malaria, diarrhoea, pneumonia and eczema were 34, 65, 10 and 5 per 100 person-years, respectively. Measles and tuberculosis were very rare in the cohort with only 1 and 4 episodes observed, respectively, so it was not possible to evaluate the impact of anthelminthic treatment on incidence of these diseases. Neither maternal albendazole nor maternal praziquantel had any effect on the incidence of malaria, diarrhoea or pneumonia overall, or in the pre-specified sub-group analyses. Maternal albendazole was associated with a higher rate of eczema overall (HR 1.58 (95% CI 1.15, 2.17), p = 0.005) but maternal praziquantel showed no such effect (HR 1.15 (95% CI 0.83, 1.58), p = 0.40; Figure 2, Table 5). The effect of maternal albendazole treatment was strong in infancy (HR 1.78 (95% CI 1.25, 2.55), p = 0.002) and a diagnosis of eczema during infancy was a strong predictor of further eczema in 1–5 year olds (HR 6.36 (95% CI 3.60, 11.22), p<0.001). After adjusting for a previous diagnosis of eczema in infancy there was little evidence of an independent effect of maternal albendazole in children aged 1 to 5 years (HR 1.20 (95% CI 0.75, 1.93), p = 0.44). The adverse effect of maternal albendazole appeared to be stronger among children of mothers without hookworm than among children of mothers with hookworm, but the interaction was not statistically significant (p = 0.15). Maternal praziquantel showed no differential effect on eczema according to maternal *S. mansoni* infection status (Table 6).

**Table 5:** The effect of anthelminthic treatment during pregnancy on incidence of malaria, diarrhoea, pneumonia and eczema during early childhood (from birth to 5 years).

		Albendazole Placebo	Albendazole	Praziquantel Placebo	Praziquantel
**Malaria**	Events (pyrs at risk ×100)	1641 (47.67)	1640 (47.78)	1617 (48.21)	1664 (47.24)
	Rate per 100 pyrs	34.43	34.32	33.54	35.22
	Hazard ratio (95% CI)		1.00 (0.88–1.13)		1.04 (0.92–1.18)
	P value		0.95		0.49
**Diarrhoea**	Events (pyrs at risk ×100)	3003 (47.14)	3127 (47.18)	3010 (47.68)	3120 (46.64)
	Rate per 100 pyrs	63.71	66.28	63.13	66.90
	Hazard ratio (95% CI)		1.04 (0.96–1.12)		1.05 (0.97–1.14)
	P value		0.34		0.22
**Pneumonia**	Events (pyrs at risk ×100)	454 (48.11)	500 (48.17)	479 (48.64)	475(47.64)
	Rate per 100 pyrs	9.44	10.38	9.85	9.97
	Hazard ratio (95% CI)		1.10 (0.92–1.31)		1.00 (0.84–1.20)
	P value		0.31		0.97
**Eczema**	Events (pyrs at risk ×100)	175 (48.21)	277 (48.26)	212 (48.74)	240 (47.73)
	Rate per 100 pyrs	3.63	5.74	4.35	5.03
	Hazard ratio (95% CI)		1.58 (1.15–2.17)		1.15 (0.83–1.58)
	P value		0.005		0.40

There was no evidence of interaction between maternal albendazole and praziquantel treatments, therefore the effects of each treatment were examined independently.

**Table 6:** The effect of maternal anthelminthic treatment on childhood disease incidence by maternal helminth status (from birth to 5 years).

		Albendazole		Praziquantel	
		Maternal hookworm	No maternal hookworm	Maternal schistosomiasis	No maternal schistosomiasis
		N = 1025	N = 1311	N = 421	N = 1915
**Malaria**	Hazard ratio for treatment effect (95% CI)	1.09 (0.92,1.30)	0.92 (0.76–1.10)	1.09 (0.91,1.30)	0.98 (0.82,1.17)
	P value for interaction	0.18		0.41	
**Diarrhoea**	Hazard ratio for treatment effect (95% CI)	1.10 (0.98,1.24)	0.99 (0.89,1.10)	1.17 (0.96,1.43)	1.03 (0.95,1.12)
	P value for interaction	0.17		0.26	
**Pneumonia**	Hazard ratio for treatment effect (95% CI)	1.11 (0.85,1.46)	1.09 (0.85,1.38)	1.21 (0.81,1.80)	0.97 (0.79,1.19)
	P value for interaction	0.90		0.33	
**Eczema**	Hazard ratio for treatment effect (95% CI)	1.15 (0.72–1.83)	1.82 (1.19–2.79)	1.64 (0.87–3.07)	1.10 (0.76–1.58)
	P value for interaction	0.15		0.28	

**Figure 3:**
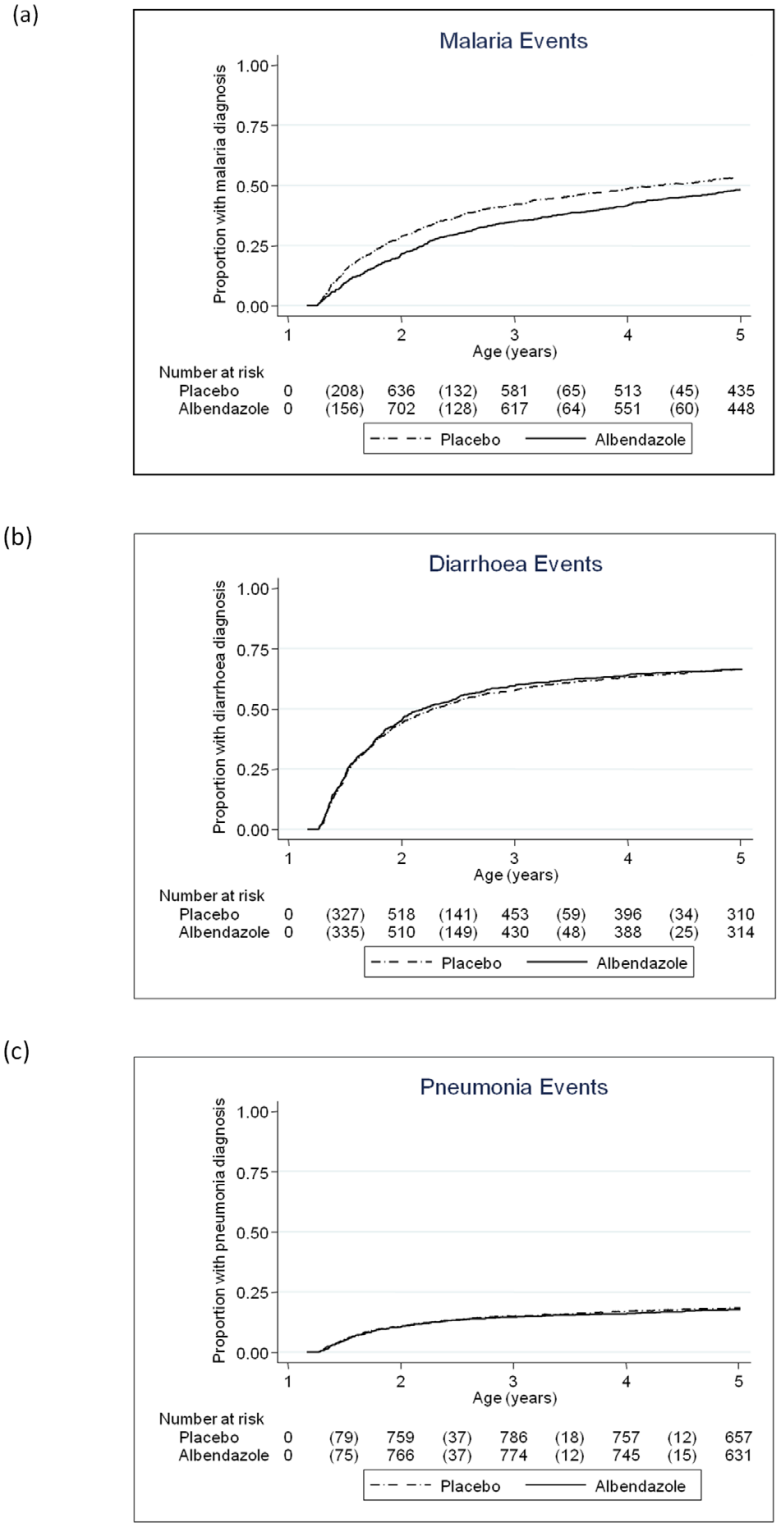
Effect of quarterly albendazole from age 15 months to 5 years on infectious disease incidence in children. Kaplan-Meier survival estimates for time to first (or only episode) of (a) malaria, (b) diarrhoea and (c) pneumonia during the intervention period, comparing children who received quarterly albendazole with those who received placebo. Numbers shown in the tables are number of events (in brackets) and number of children at risk.

Childhood quarterly albendazole was associated with reduced malaria incidence (HR 0.85 (95% CI 0.73, 0.98), p = 0.03; Table 7), and a post-hoc subgroup analysis found that this effect was strongest in the second year of life (interaction p = 0.002, Figure 3).This trend was replicated in the prevalence of asymptomatic *P. falciparum* parasitaemia at 2 years (Table 8). There was no effect of childhood quarterly albendazole on pneumonia, diarrhoea or eczema (Table 7).

**Table 7:** The effect of quarterly albendazole during childhood on incidence of malaria, diarrhoea, pneumonia, and eczema (15 months to 5 years).

		Albendazole Placebo	Albendazole
**Malaria**	Events (pyrs at risk ×100)	1006 (31.67)	845 (31.11)
	Rate per 100 pyrs	31.77	27.16
	Hazard ratio (95% CI)		0.85 (0.73–0.98)
	P value		0.03
**Diarrhoea**	Events (pyrs at risk ×100)	1173 (31.59)	1147 (30.97)
	Rate per 100 pyrs	37.13	37.04
	Hazard ratio (95% CI)		0.99 (0.88–1.11)
	P value		0.84
**Pneumonia**	Events (pyrs at risk ×100)	211 (31.97)	206 (31.35)
	Rate per 100 pyrs	6.60	6.57
	Hazard ratio (95% CI)		0.99 (0.76–1.28)
	P value		0.92
**Eczema**	Events (pyrs at risk ×100)	77 (32.03)	107 (31.39)
	Rate per 100 pyrs	2.40	3.41
	Hazard ratio (95% CI)		1.25 (0.78–2.01)*
	P value		0.36

* Hazard Ratio adjusted for eczema prior to randomisation and maternal hookworm at enrolment.

**Table 8:** The effect of quarterly albendazole during childhood on asymptomatic malaria parasitaemia, haemoglobin and growth.

	2 years		3 years		4 years		5 years		Repeated measures analysis	
	Placebo	Albendazole	Placebo	Albendazole	Placebo	Albendazole	Placebo	Albendazole	Effect measure	p-value^a^
	(n = 745)	(n = 722)	(n = 759)	(n = 751)	(n = 743)	(n = 707)	(n = 726)	(n = 697)	(95% CI)^a^	
**Asymptomatic malaria parasitaemia**										
**Positive (%)**	59 (8.1)	30 (4.3)	32 (4.3)	34 (4.7)	32 (4.5)	30 (4.4)	36 (5.1)	33 (4.9)		
**OR**		0.51		1.09		0.99		0.95		
**(95% CI)**		(0.32–0.80)		(0.66–1.78)		(0.59–1.64)		(0.59–1.55)		
**Haemoglobin**										
**Mean (SD)**	11.06 (1.27)	11.09 (1.39)	11.78 (1.14)	11.87 (1.12)	12.14 (1.00)	12.11 (1.13)	12.16 (1.20)	12.09 (1.19)		
**Difference**		0.02		0.08		−0.02		−0.06	0.01	0.91
**(95% CI)**		(−0.11, 0.16)		(−0.33, 0.20)		(−0.17, 0.12)		(−0.19, 0.06)	(−0.08, 0.09)	
**Weight-for-age z-score**										
**Mean (SD)**	−0.57 (1.07)	−0.61 (1.09)	−0.66 (1.05)	−0.67 (1.00)	−0.77 (0.97)	−0.81 (0.99)	−0.87 (0.91)	−0.88 (0.95)		
**Difference**		−0.04		−0.00		−0.04		0.01	−0.02	0.72
**(95% CI)**		(−0.15, 0.07)		(−0.11, 0.10)		(−0.14, 0.06)		(−0.11, 0.09)	(−0.10, 0.07)	
**Height-for-age z-score**										
**Mean (SD)**	−0.98 (1.37)	−0.88 (1.25)	−0.77 (1.12)	−0.72 (1.22)	−0.90 (1.26)	−0.87 (1.22)	−1.27 (1.20)	−1.33 (1.34)		
**Difference**		0.09		0.06		0.03		−0.06	0.03	0.58
**(95% CI)**		(−0.04, 0.23)		(−0.06, 0.18)		(−0.10, 0.15)		(−0.19, 0.07)	(−0.07,0.13)	
**Weight-for-height z-score** a										
**Mean (SD)**	−0.12 (1.39)	−0.22 (1.33)	−0.36 (1.18)	−0.43 (1.13)	−0.36 (1.21)	−0.45 (1.17)	−0.17 (1.19)	−0.13 (1.28)		
**Difference**		−0.10		−0.07		−0.08		0.04	−0.05	0.25
**(95% CI)**		(−0.24, 0.04)		(−0.19, 0.06)		(−0.20, 0.04)		(−0.10, 0.18)	(−0.14, 0.04)	

Malaria parasitaemia results were missing for 39, 53, 52 and 38 children at ages 2, 3, 4 and 5 years, respectively; haemoglobin results were missing for 17, 28, 658^b^ and 13 children at ages 2, 3, 4 and 5 years, respectively; weight-for-age z-scores were missing for 2, 0, 1 and 4 children at ages 2, 3, 4 and 5 years, respectively; height-for-age z-scores were missing for 12, 2, 9 and 12 children at ages 2, 3, 4 and 5 years, respectively; weight-for-height z-scores were missing for 16, 4, 11 and 184^c^ children at ages 2, 3, 4 and 5 years, respectively.

a The effect of quarterly albendazole on asymptomatic malaria parasitaemia changed with time (interaction p = 0.02), therefore the overall effect of the intervention on this outcome is not presented.

b Haemoglobin was not measured for four-year olds from 22^nd^ January 2009 onwards due to budget constraints.

c Weight-for-height z-scores could not be calculated using WHO Anthro software for children who were aged >5 years and 1 month.

There was no effect of maternal anthelminthic treatment overall, or in pre-specified sub-group analyses, on childhood haemoglobin levels or growth (data not shown). Additionally there were no effects of childhood quarterly albendazole on these parameters either when examining each annual visit separately (Table 8), or when data were combined and analysed using GEE regression models.

At age 5 years, 870 participants were assessed on verbal and nonverbal cognitive abilities, including executive function and working memory, as well as motor abilities. Albendazole treatment during pregnancy was associated with a marginally lower score on one measure of executive function (the Wisconsin card-sorting test: regression coefficient −0.54 (95% CI −1.07, −0.01; p = 0.05)); praziquantel treatment was associated with higher score on a gross motor function, balancing on one leg (regression coefficient 1.58 (95%CI 0.04, 3.11; p = 0.04). No effects were observed in the pre-specified subgroup analyses. There was no effect of childhood quarterly albendazole on any score (Table S4).

Serious adverse events during pregnancy and infancy have been described. [30], [31] Twenty-nine deaths occurred during childhood. There were no associations between maternal anthelminthic treatments and mortality rate. One child developed severe malaria within three days of receiving their first dose of albendazole at age 17 months, and died 6 weeks later following complications from this illness event, but there was no suggestion of a consistent effect of the childhood intervention on mortality: only 16 deaths occurred after the childhood randomisation, 8 in the placebo group and 8 in the albendazole group.

## Discussion

In Entebbe, Uganda, where helminth prevalence was high among pregnant women, we addressed the effects of treatment with albendazole and praziquantel during the second and third trimesters of pregnancy on outcomes from birth to 5 years. We found no substantial effect of anthelminthic treatment during pregnancy on the child's response to immunogens, on infectious disease incidence, or on anaemia, growth, motor or cognitive development, to age 5 years. By contrast, treatment with albendazole during pregnancy had an adverse effect on the incidence of eczema in childhood. These longer-term results accord with our findings in infancy [15], [16]. Helminth infection prevalence was unexpectedly low during early childhood, and this limited our ability to assess the effects of the childhood intervention against them. However, we found that quarterly albendazole from age 15 months to 5 years reduced malaria incidence, with strongest effect between age 15 months and 2 years.

Many studies on the effects of helminths and their treatment are flawed due to bias and confounding, because of strong associations between helminth infections and poverty, deprivation and poor health care. A strength of this study was its randomised, placebo-controlled design, which resulted in the balanced distribution of many potential confounding factors between the treatment arms. Helminth prevalence during pregnancy was high: the detection of at least one species among 68% of women using a single stool sample implies that almost all women were infected. [32], [33] By contrast, the low prevalence of helminth infection among children was our chief limitation. For ethical reasons, effective treatment was provided annually to children found to be helminth-infected; therefore annual prevalence figures represent not cumulative infection since birth, but cumulative incidence since the most recent treatment. Although quarterly albendazole reduced infection rates with *Ascaris* and hookworm, the numbers infected were so small that any consequence of this reduction would have had to have been very strong to be detected in this study. Questions as to the effects of helminth infection and their regular treatment in preschool children therefore remain unanswered. A possible bias is suggested by the somewhat higher uptake of the childhood intervention by children receiving placebo, compared to those receiving active drug. However, we had no evidence that either staff or participants had inadvertently been unblinded to treatment allocation. Mothers of children who participated in the childhood intervention differed from mothers of children who did not. This is unlikely to have biased our treatment effect estimates since characteristics of participants were balanced between treatment groups. However it may have reduced our power to detect effects of anthelminthic treatment since the participants who were lost tended to have higher prevalence of some helminths, and could have some implications for generalisability since children who participated in the childhood intervention were on average from slightly more well-off families within the study setting. We examined the effects of three interventions, each on multiple outcomes; all analyses except for one exploratory outcome (asymptomatic malaria parasitaemia) were pre-specified, however the possibility that our positive findings are due to chance alone cannot be discounted. Rather than formally adjusting for multiple testing we interpret consistent results for related outcomes as providing stronger evidence of a true treatment effect.

In keeping with results in infancy, we found no effect of maternal anthelminthic treatment on recall responses to vaccine antigens at age 5 years. Quarterly albendazole during childhood was associated with reductions in type one and type two immune responses to cCFP, but this was contrary to the predicted effect of removing either worms or malaria and was not reflected by responses to antigen 85, so these rather weak effects may have arisen by chance.

We have previously reported that albendazole treatment during pregnancy was associated with increased incidence of infantile eczema, while praziquantel was associated with increased infantile eczema among the offspring of mothers with schistosomiasis, and that infantile eczema was associated with skin prick test positivity to common allergens in this cohort. [16] Eczema incidence declined, as expected, after infancy and we now show that the incidence of newly diagnosed eczema after age 1 year was similar between maternal treatment groups: thus the principal impact of maternal albendazole was in infancy. The effect of praziquantel treatment during pregnancy among infants of mothers with schistosomiasis [16] waned with the longer follow-up period. Eczema in infancy, however, remained a strong predictor of subsequent episodes, and the long term impact of intrauterine exposure on allergy-related outcomes such as asthma remains to be determined. The effect of maternal albendazole was as strong among children of women without hookworm (and indeed among women without any helminth infection) as among children of hookworm-infected women, implying a mechanism involving effects on other organisms (such as malaria, as discussed below), or a direct effect on the developing immune system. [16] The lack of effect of our childhood intervention on eczema – unintentionally tested largely in the absence of helminths – suggests that this adverse effect of albendazole was confined to the prenatal period, in keeping with studies suggesting that prenatal exposures are critical in the programming of allergy-related disease. [34]

Given the low prevalence of helminths in childhood, we were surprised by the effect of childhood albendazole on malaria incidence. This seems unlikely to be a chance finding, because illness events and asymptomatic parasitaemia showed the same pattern. Direct inhibition of malaria by benzimidazoles, including albendazole, has been demonstrated in vitro, [35] but effects in vivo differ between animal models. [36] The effect was predominantly seen between age 1 and 2 years, in the vulnerable period when maternal immunity has waned and individual immunity has not yet been established: but this does not accord with the general experience that antimalarial drugs have greater benefit among individuals with good immunity than among those with poor immunity. [37] Thus the potential contribution of regular albendazole treatment to the control of malaria in young children merits further investigation in trials designed with this as the primary outcome. The observed effect of childhood albendazole on malaria also has implications for the interpretation of other studies addressing effects of benzimidazole anthelminthics on growth, anaemia and mortality: outcomes for which malaria is likely to have a more potent impact than helminths. For example, Alderman and colleagues found benefits of albendazole for growth when given to preschool children on child health days in malaria-endemic regions of Uganda. [38] Similarly, the findings complicate the interpretation of trials intended to assess the immunological effects of removing helminths on susceptibility to malaria, such as that recently reported from Nigeria, in which regular provision of albendazole in preschool children reduced the prevalence of *Ascaris* and attenuated the increase in malaria prevalence that occurred over time. [39]

Our findings raise concerns regarding the policy of routine “deworming” with albendazole during pregnancy in developing countries. Our study commenced in a setting of high helminth prevalence, but low intensity. This picture has changed rapidly in Entebbe, as in many towns across sub-Saharan Africa, with rapid development and urbanisation over the last two decades: over 30% of Africans are now estimated to be “middle class”. [40] These demographic changes are accompanied by epidemiological transition with non-communicable diseases, including allergy-related conditions, [41] emerging as important health issues. While routine anthelminthic treatment during pregnancy may be acceptable in rural settings where hookworm infection is still high, we believe it should be avoided in urban settings where helminth infections are now low. Conversely, we found no statistically significant adverse effect of albendazole in preschool children, and large controlled trials suggest little impact of routine anthelminthic treatment among school children on allergy-related disease, [42], [43] so it seems reasonable for on-going mass treatment of worms to continue in these age groups, even as helminth prevalence becomes marginal. Regular albendazole treatment in preschool children may have an additional benefit for malaria control, especially in areas where helminths and malaria are co-endemic, and where IPTi is hampered because SP resistance is high.

## Supporting Information

Text S1 **Methods for assessment of motor and cognitive functioning at age five years.**
(DOCX)

Table S1 **Measures of motor and cognitive ability used for assessments at age five years.**
(DOCX)

Table S2 **Baseline characteristics of mothers enrolled in the factorial trial of anthelminthic treatment during pregnancy.**
(DOCX)

Table S3 **The overall prevalence of helminth infection at each routine annual visit.**
(DOCX)

Table S4 **The effect of quarterly albendazole during childhood on cognitive and motor development scores at age 5 years.**
(DOCX)

Protocol S1 **Trial Protocol.**
(PDF)

Checklist S1 **CONSORT Checklist.**
(DOC)
