# Supplementary material for: Impact of Anthelminthic Treatment in Pregnancy and Childhood on Immunisations, Infections and Eczema in Childhood: A Randomised Controlled Trial
Source: PLoS One. 2012 Dec 7;7(12):e50325. doi: 10.1371/journal.pone.0050325 (PMC3517620; doi:10.1371/journal.pone.0050325)
Supplement: Text S1 — Methods for assessment of motor and cognitive functioning at age five years. (DOCX) [file pone.0050325.s001.docx]

**Text S1: Methods for assessment of motor and cognitive functioning at age five years**

Motor and cognitive ability at age five years was assessed using measures adapted from Western tools to be appropriate for Ugandan children, as previously described [1]. The full battery of measures used, and the domains that they assessed, are shown in Table S1. Four of these measures were not described in our earlier work, and are described here, as follows.

*Testing Procedure*

Motor and cognitive assessments were administered at the study clinic by a team of nurses and a doctor trained to administer the various measures. Sessions lasted 60-90 minutes including short breaks. Testing was postponed for children who were sick provided it was not longer than two months past the target test date. Children received an age-appropriate gift in appreciation for their participation and transport costs were reimbursed to the parent.

*Description of tests not previously described*

*Counting Span.* The task was originally developed by Case and colleagues in 1982 [2], to measure total processing and storage space (*M-space*) for working memory in children aged 6-12 years. A simpler modification of the counting span task was constructed and used to assess working memory in our participants. It consists of eight arrays of cards which the child counts and has to note the picture on the target card (first card). At the end of counting the cards in each array, the child is required to recall and say the picture on the target card. The first trial comprised an array of three cards, the second trial four cards and so on up to the eighth trial which had ten cards. The pictures used were all locally hand drawn black-and-white line drawings of relatively equal size. The experimenter initially reviewed all the pictures on the cards with the child to ensure that the child knew all their names. Trials were preceded by a demonstration example. One point was awarded for a correct response and a zero for an incorrect response and feedback was given for each trial done. There was therefore a maximum possible score of eight on this measure.

*Running Memory.* This measure was adapted from the running memory task version of Kramer and colleagues [3] to measure working memory span. The version devised for this study consisted of 10 strings of common, unrelated two-syllable Luganda words, which are read out to the child at a regular speed of one word per two seconds. The child was required to repeat each string verbatim immediately without changing the word order, omitting or inserting a word. Two points were awarded if no error was committed; one point if not more than two errors were committed; and no points (zero) if three or more errors were committed on a particular string.

*Shapes Task.* This measure was originally developed by Kochanska and colleagues [4] utilizing the Stroop paradigm [5] to measure inhibition. A modification of the Shapes Task was constructed to assess inhibitory control of children participating in this study. This consists of 24 8x11inch pictures representing large shapes (animals, fruits, household items). Each shape is cut out of paper covered with a design that depicts much smaller shapes. Geometric figures, numbers and letters present in the original version were excluded since most of the children have not yet learnt these concepts at the age of five years. In 12 trials (*consistent*), the small shapes were consistent with the large shape (e.g. a large moon made up of small moons) and in the other 12 trials (*inconsistent*) the shapes were inconsistent (e.g. a large cat made up of small bunnies or a large cow made up of small balls). The experimenter initially reviewed all of the shapes with the child to ensure that he/she knew their names. The pictures were then presented one by one, and the child was to name as fast as possible the small shape in each picture. To prime the child’s bias to attend to the global shape rather than the smaller design, the inconsistent pictures were interspersed with the consistent ones. Scoring was done concurrently and scores were a pass for a correct response and a zero for an incorrect response. Scores on the 12 inconsistent trials were used for analysis.

*Tower of London.* This measure was adapted from Shallice’s version of Tower of London (TOL) [6] to test planning or problem solving. The task requires moving differently coloured balls across three equal sized pegs in order to duplicate a pre-specified target configuration. Three constraints apply: the child must not place more than the permitted number of balls on one peg, must not place the balls anywhere other than the peg, and only one ball is moved at a time. The TOL used in this sample comprised 10 trials altogether. In five of the trials, a single tower configuration was presented as the target. In the other five trials mixed configurations were presented in which the design to be duplicated was spread over two or more pegs. The single tower configurations were alternated with the mixed configuration trials. The first four trials required three moves, the next four trials four moves and the last two trials required five moves. Performance was judged based on the number of correct configurations, and the number of moves taken to arrive at the target configuration.

**References**

1. Nampijja M, Apule B, Lule S, Akurut H, Muhangi L, et al. (2009) Adaptation of Western measures of cognition for assessing 5 year-old semi-urban Ugandan children. Br J Educ Psychol.

2. Case R, Kurland MD, Goldenberg J (1982) Operational efficiency and the growth of short- term memory span. Journal of Experimental Child Psychology 33: 386-404.

3. Kramer AF, Larish JF, Strayer DL (1995) Training for attentional control in dual task settings: A comparison of young and old adults. . Journal of Experimental Psychology: (Applied) 1: 50-76.

4. Kochanska G, Murray K, Coy KC (1997) Inhibitory control as a contributor to conscience in childhood: From toddler to early school age. . Child Development 68: 263-277.

5. Rothbart MK, Derryberry D, Posner MI (1994) A psychological approach to the development of temperament. In: Bates JE, Wachs TD, editors. Temperament: Individual differences at the interface of biology and behaviour Washington, DC: American Psychological Association. pp. 83-116.

6. Shallice T (1982) Specific impairments of planning. Philos Trans R Soc Lond B Biol Sci 298: 199-209.
