# Supplementary material for: Impact of Anthelminthic Treatment in Pregnancy and Childhood on Immunisations, Infections and Eczema in Childhood: A Randomised Controlled Trial
Source: PLoS One. 2012 Dec 7;7(12):e50325. doi: 10.1371/journal.pone.0050325 (PMC3517620; doi:10.1371/journal.pone.0050325)
Supplement: Table S1 — Measures of motor and cognitive ability used for assessments at age five years. (DOCX) [file pone.0050325.s002.docx]

**Table S1. Measures of motor and cognitive ability used for assessments at age five years**

| **Domain tested** | **Measure** | |
| --- | --- | --- |
| General cognitive ability | 1 | Block design |
|  | 2 | Picture vocabulary scale |
| Measures of working memory | 3 | Sentence repetition |
|  | 4 | Verbal fluency |
|  | 5 | Counting span |
|  | 6 | Running memory |
| Measures of attention | 7 | Picture search |
| Measures of cognitive flexibility | 8 | Wisconsin card sort test |
| Measures of inhibition | 9 | Tap once tap twice task |
|  | 10 | Shapes task |
| Measures of planning | 11 | Tower of London |
| Measures of fine motor function | 12 | Coin box |
| Measures of gross motor function | 13 | Balancing on one leg |
