# Supplementary material for: Impact of Anthelminthic Treatment in Pregnancy and Childhood on Immunisations, Infections and Eczema in Childhood: A Randomised Controlled Trial
Source: PLoS One. 2012 Dec 7;7(12):e50325. doi: 10.1371/journal.pone.0050325 (PMC3517620; doi:10.1371/journal.pone.0050325)
Supplement: Table S2 — Baseline characteristics of mothers enrolled in the factorial trial of anthelminthic treatment during pregnancy. (DOCX) [file pone.0050325.s003.docx]

**Table S2. Baseline characteristics of mothers enrolled in the factorial trial of anthelminthic treatment during pregnancy**

|  | **Albendazole + praziquantel** | **Praziquantel only** | **Albendazole only** | **Placebo** |
| --- | --- | --- | --- | --- |
| Number enrolled | 628 | 626 | 625 | 628 |
| Age in years, mean ± SD | 23.97 ± 5.50 | 23.52 ± 5.41 | 23.49 ± 5.30 | 23.34 ± 5.17 |
| Education (4 mv)^b^ |  |  |  |  |
| None | 25 (4%) | 23 (4%) | 17 (3%) | 32 (5%) |
| Primary | 311 (50%) | 318 (51%) | 318 (51%) | 316 (50%) |
| Secondary | 235 (37%) | 229 (37%) | 244 (39%) | 226 (36%) |
| Tertiary | 56 (9%) | 55 (9%) | 45 (7%) | 53 (8%) |
| Household socioeconomic status (49 mv) ^c^ |  |  |  |  |
| (low) 1 | 33 (5%) | 40 (7%) | 35 (6%) | 39 (6%) |
| 2 | 54 (9%) | 57 (9%) | 54 (9%) | 52 (8%) |
| 3 | 190 (31%) | 199 (33%) | 182 (30%) | 194 (32%) |
| 4 | 174 (28%) | 174 (28%) | 179 (29%) | 183 (30%) |
| 5 | 130 (21%) | 109 (18%) | 131 (21%) | 115 (19%) |
| (high) 6 | 37 (6%) | 32 (5%) | 33 (5%) | 32 (5%) |
| Gravidity |  |  |  |  |
| 1 | 178 (28%) | 172 (27%) | 181 (29%) | 164 (26%) |
| 2-4 | 339 (54%) | 354 (57%) | 350 (56%) | 369 (59%) |
| ≥5 | 111 (18%) | 100 (16%) | 94 (15%) | 95 (15%) |
| Trimester at treatment (3mv) |  |  |  |  |
| 2 | 338 (54%) | 325 (52%) | 321 (51%) | 325 (52%) |
| 3 | 290 (46%) | 300 (48%) | 304 (49%) | 301 (48%) |
| Helminth infections |  |  |  |  |
| Hookworm (11 mv) | 270 (43%) | 301 (48%) | 262 (42%) | 277 (44%) |
| *S. mansoni* (11 mv) | 117 (19%) | 104 (17%) | 123 (20%) | 114 (18%) |
| *M. perstans* (8 mv) | 135 (22%) | 136 (22%) | 117 (19%) | 143 (23%) |
|  |  |  |  |  |
| HIV positive | 79 (13%) | 61 (10%) | 71 (11%) | 88 (14%) |
|  |  |  |  |  |
| Malaria parasitaemia (48 mv) | 61 (10%) | 59 (10%) | 63 (10%) | 85 (14%) |
|  |  |  |  |  |
| Owns mosquito net (4 mv) | 291 (46%) | 294 (47%) | 324 (52%) | 331 (53%) |
|  |  |  |  |  |

^a^ As previously reported (Ndibazza et al 2010, Webb et al 2011).

^b^ mv: missing values

^c^ Household socioeconomic status was scored based on building materials of the home, number of rooms and items owned, “1” representing lowest and “6” representing highest status
