# Supplementary material for: Impact of Anthelminthic Treatment in Pregnancy and Childhood on Immunisations, Infections and Eczema in Childhood: A Randomised Controlled Trial
Source: PLoS One. 2012 Dec 7;7(12):e50325. doi: 10.1371/journal.pone.0050325 (PMC3517620; doi:10.1371/journal.pone.0050325)
Supplement: Table S3 — The overall prevalence of helminth infection at each routine annual visit. (DOCX) [file pone.0050325.s004.docx]

**Table S3. The overall prevalence of helminth infection at each routine annual visit**

|  | **Age** | | | | |
| --- | --- | --- | --- | --- | --- |
|  | **1 year** | **2 years** | **3 years** | **4 years** | **5 years** |
| **Helminth** | **(n=1520)** | **(n=1527)** | **(n=1449)** | **(n=1374)** | **(n=1322)** |
| *Trichuris trichiura* | 15 (1.0%) | 30 (2.0%) | 64 (4.4%) | 75 (5.5%) | 73 (5.5%) |
| *Ascaris lumbricoides* | 21 (1.4%) | 22 (1.4%) | 25 (1.7%) | 17 (1.2%) | 14 (1.1%) |
| *Schistosoma mansoni* | 1 (0.1%) | 10 (0.7%) | 12 (0.8%) | 19 (1.4%) | 30 (2.3%) |
| Hookworm | 4 (0.3%) | 10 (0.7%) | 8 (0.6%) | 16 (1.2%) | 6 (0.5%) |
| *Hymenolepis nana* | 0 (0%) | 7 (0.5%) | 14 (1.0%) | 18 (1.3%) | 13 (1.0%) |
| *Mansonella perstans* | 8 (0.5%) | 3 (0.2%) | 5 (0.3%) | 5 (0.4%) | 3 (0.2%) |
| *Trichostrongylus* | 6 (0.4%) | 3 (0.2%) | 1 (0.1%) | 1 (0.1%) | 0 (0%) |
