# Supplementary material for: Impact of Anthelminthic Treatment in Pregnancy and Childhood on Immunisations, Infections and Eczema in Childhood: A Randomised Controlled Trial
Source: PLoS One. 2012 Dec 7;7(12):e50325. doi: 10.1371/journal.pone.0050325 (PMC3517620; doi:10.1371/journal.pone.0050325)
Supplement: Table S4 — The effect of quarterly albendazole during childhood on cognitive and motor development scores at age 5 years. (DOCX) [file pone.0050325.s005.docx]

**Table S4. The effect of quarterly albendazole during childhood on cognitive and motor development scores at age 5 years.**

| **Domain tested** | **Measure** | | **Mean test score** | | **Mean difference in test score (95% CI)** | **P value** |
| --- | --- | --- | --- | --- | --- | --- |
|  |  | | **Placebo** | **Albendazole** |  |  |
|  |  | | **n=420** | **n=440** |  |  |
| General cognitive ability | | |  |  |  |  |
|  | 1 | Block Design | 8.99 | 8.75 | -.24 (-.71, .23) | .318 |
|  | 2 | Picture Vocabulary Scale | 17.65 | 17.53 | -.13 (-.56, .31) | .576 |
| Measures of working memory | | | | | | |
|  | 3 | Sentence Repetition | 19.81 | 20.06 | .25 (-.37, .87) | .421 |
|  | 4 | Verbal Fluency | 14.02 | 14.26 | .24 (-.90, 1.39) | .675 |
|  | 5 | Counting Span* | 3.68 | 3.46 | -.22 (-.86, .42) | .503 |
|  | 6 | Running Memory* | 12.35 | 11.94 | -.41 (-1.25, .43) | .341 |
| Measure of attention | | |  |  |  |  |
|  | 7 | Picture Search | 3.98 | 4.05 | .07 (-.13, .26) | .499 |
| Measure of cognitive flexibility | | |  |  |  |  |
|  | 8 | Wisconsin Card Sort Test | 6.34 | 6.40 | .06 (-.49, .61) | .834 |
| Measures of inhibition | | |  |  |  |  |
|  | 9 | Tap Once Tap Twice | 5.90 | 6.23 | .33 (-.31, .98) | .310 |
|  | 10 | Shapes Task* | 6.51 | 5.34 | -1.17 (-2.37, .04) | .058 |
| Measure of planning | | |  |  |  |  |
|  | 11 | Tower of London* | 2.58 | 2.26 | -.31 (-1.27, .64) | .518 |
| Measure of fine motor function | | |  |  |  |  |
|  | 12 | Coin Box | 9.86 | 9.97 | .11 (-.17, .39) | .428 |
| Measure of gross motor function | | |  |  |  |  |
|  | 13 | Balancing on One Leg | 15.00 | 15.41 | .41 (-1.17, 1.99) | .609 |

* Tests conducted only in a subset of cases: n(Placebo)=81; n(Albendazole) = 89
